# Evaluation of Central Macular Thickness and Retinal Nerve Fiber Layer Thickness using Spectral Domain Optical Coherence Tomography in a Tertiary Care Hospital

**DOI:** 10.5005/jp-journals-10008-1165

**Published:** 2014-06-12

**Authors:** Prakashchand Agarwal, VK Saini, Saroj Gupta, Anjali Sharma

**Affiliations:** Assistant Professor, Department of Ophthalmology, People's College of Medical Sciences and Research Centre, Bhopal, Madhya Pradesh, India; Professor and Head, Department of Ophthalmology, People's College of Medical Sciences and Research Centre, Bhopal, Madhya Pradesh, India; Professor, Department of Ophthalmology, People's College of Medical Sciences and Research Centre, Bhopal, Madhya Pradesh, India; Senior Resident, Department of Ophthalmology, People's College of Medical Sciences and Research Centre, Bhopal, Madhya Pradesh, India

**Keywords:** Spectral Domain OCT, Normative data, Central India.

## Abstract

**Purpose:** To evaluate the normative data of macular thickness and retinal nerve fiber layer thickness (RNFL) among normal subjects using spectral domain optical coherence tomography (OCT).

**Materials and methods:** Normal subjects presenting to a tertiary medical hospital were included in the study. All patient underwent clinical examination followed by study of macular thickness and RN FL thick ness by spectral domain Topc on OCT. The data was collected and analyzed for variations in gender and age. The data was also compared with available literature.

**Results:** Total numbers of patients enrolled in the study were 154 (308 eyes). Numbers of males were 79 (158 eyes) and numbers of females were 75 (150 eyes). The mean age among males was 42.67 ± 12.15 years and mean age among females was 42.88 ± 11.73 years.

Overall the mean mac ular thickness (central 1 mm zone) with SD - OCT was 241.75 ± 17.3 microns. The mean macular volume was 7.6 cu. mm ± 0.33. On analysis of the RNFL thickness, we observed that the RNFL was thickest in the inferior quadrant (138.58) followed by superior (122.30) nasal (116.32) and temporal quadrant (73.04).

Gender-wise comparison of the data revealed no statistically significant difference for age, macular thickness parameters, volume and RFNL values except outer temporal thickness among males and females. No age-related difference was noted in the above parameters. On comparison with available norma tive data from India and elsewhere, we found significant variations with different machines.

**Conclusion:** The study is the first to provide normative data using SD-OCT from central India. The data from spectral domain OCT correlated well with the values obtained from similar studies with SD - OCT. Values obtained from time domain OCT machines are different and are not comparable.

**How to cite this article:** Agarwal P, Saini VK, Gupta S, Sharma A. Evaluation of Central Macular Thickness and Retinal Nerve Fiber Layer Thickness using Spectral Domain Optical

Coherence Tomography in a Tertiary Care Hospital. J Curr Glaucoma Pract 2014;8(2):75-81.

## INTRODUCTION

Optical coherence tomography is a standard non invasive diagnostic test today to visualize the morphology of retina. It provides high-resolution, cross-sectional, quanti tative image of the retina and helps us measure the thickness of retina at various points. Central macular thickness can be measured with the OCT and correlated with clinical exami nation and visual function.^1,2^ Similarly, retinal nerve fiber layer thickness (RFNL) around the disc (peripapillary RFNL) can be measured with the OCT and correlated with the health of neural retinal rim of the optic nerve head and visual fields of the patient.^3,4^ With evolution and refinement of technology now we have moved from time domain to spectral domain OCT. This has lesser image acquisition time and provides high resolution images which help us delineate pathology from normal tissues.^5-8^ There are very few large studies on the normative data for macular thickness using the spectral OCT. The macular thickness measurement for diagnostic function may differ with the population used as a database. There are differences in normative data with respect to age, sex, gender and race.^9,10^ Such differences need to be taken into account while interpreting raw data. Most of the newer generation machines have inbuilt normative data and hence, are able to differentiate normal values from abnormal and represent it in a color coded manner. However, apart from color coding representation of data; knowledge of normal absolute values is also essential which may vary between different machines. The absolute cut-off values of central macular thickness may be a deciding factor to treat the macular edema, which may vary according to the machine being used. Similarly, in certain scenarios absolute values of RFNL may be deciding factor in diagnosis of glaucoma.^11,13^ Thus, it is essential for the operator and ophthalmologist to have complete knowledge of normative data of the machine being used to examine the respective patient. Most of the studies of normative data of macular thickness and retinal nerve fiber thickness (RFNL) were done in northern^14,15^ or southern^16,17^ India using time domain OCT. This study was done to establish the normal macular thickness and RFNL para meters using spectral domain OCT (3D OCT 2000, Topcon corporation, Tokyo, Japan) in central India at a tertiary medical college.

## AIM

To evaluate the central macular thickness (CMT) and retinal nerve fiber layer (RFNL) thickness in normal subjects presenting at tertiary care hospital using spectral domain optical coherence tomography (OCT).

## MATERIALS AND METHODS

### Materials

Our study was conducted at ophthalmology department and healthy volunteers presenting to eye out patient department were included in this cross-sectional study. This study was approved from the research and ethics committee of the institute. Informed consent was obtained. All subjects underwent vision, refraction, examination of eye with slit-lamp, Goldmann applanation tonometry, gonioscopy and fundus examination with plus 90D lens.

Inclusion criteria were age > 18 years, healthy volunteers consti tuting attendants of patients, hospital staff, contralateral normal eye of patients were included in this cross-sectional study. Exclusion criteria were family history of glaucoma, history of prior photocoagulation, history of prior ocular disease, history of intraocular surgery, previous ocular t rauma, vertical asymmetry of cup: disk (C:D) ratio (>0.2) between the two eyes, high C: D ratio (>0.6), disk hemorrhages, disk pallor, and localized RNFL defects, refractive error of > ±4 diopter, intraocular pressure >22 mm Hg. Optical coherence tomography was performed using 3D OCT 2000 (Topcon corporation, Tokyo, Japan), with software version 3.

### Methods of Evaluation

Eyes that fulflled both exclusion and inclusion criteria were selected for analysis, if both eyes fulflled the criteria, both the eyes were included. After complete clinical examination, each eye was dilated with tropicamide 1% before recording the images, and scans were performed with a minimum pupillary diameter of 5 mm. After entry of details of patient inclu di ng age, sex, r ace (Asian) specific examination modes were selected.

Central Macular Thickness

The macular evaluation mode was selected from the computer console. The scan was performed with 3D 6.0 × 6.0 pro tocol. The image was taken with green cross as the internal fixation target. After saving the computer image the analyzed data values using inbuilt protocol was noted. The report generated by the machine gives the color image of central macular with image centered at the fovea. The macular thickness is depicted as concentric circles of 1, 3, and 6 mm from the center of fovea. All the values of macular thickness and macular volume were noted, tabulated and analyzed.

For RFNL Analysis

The glaucoma evaluation mode was selected from the computer console. The scan was performed with 3D 6.0 × 6.0 protocol. The image was taken with green cross as the inter nal fixation target. After saving the computer image the analyzed data values using inbuilt protocol was noted. The report generated by the machine gives the color image of optic nerve head surrounded by 3.4 mm green centration ring. It gives the peripapillary RFNL thickness of superior, inferior, nasal and temporal quadrants along with total average RFNL thickness. All these values were noted and analyzed.

## RESULTS

Total numbers of patients enrolled in the study were 154 (308 eyes). Numbers of males were 79 (158 eyes) and numbers of females were 75 (150 eyes). The mean age among males was 42.67 ± 12.15 years and mean age among females was 42.88 ± 11.73 years.

Overall, the mean macular thickness (central 1 mm zone) with SD-OCT was 241.75 ± 17.3 microns. The mean macular volume was 7.6 ± 0.33 cu. mm. On analysis of the RNFL thickness, we observed that the RNFL was thickest in the inferior quadrant (138.58) followed by superior (122.30) nasal (116.32) and temporal quadrant (73.04) (Table 1).

Gender-wise comparison of the data revealed no statis tically significant difference age, macular thickness parameters, volume and RFNL values except outer temporal thickness (OTT) among males and females (Table 2). The standard deviation (SD) of OTT among males is 5.35 and among females is 10.21. This difference of SD is responsible for statistical significance among the two groups.

To study age-related change in macular thickness and RNFL values, the data was divided into two groups with age < 40 years (152 eyes) and second group age ≤ 40 years (156 eyes). No statistically significant difference was noted in macular thickness parameters, volume, and RFNL values (Table 3).

**Table 1:** **Table 1:** Normative data of OCT parameters of 154 subjects (308 eyes)

		*N*		*Minimum*		*Maximum*		*Mean*		*Std. deviation*	
Average macular thickness		154 (308 eyes)		269.00		295.00		279.4277		7.17867	
Outer nasal thickness		154 (308 eyes)		261.00		308.00		283.7143		12.94570	
Outer temporal thickness		154 (308 eyes)		244.00		286.00		253.6039		8.34659	
Outer superior thickness		154 (308 eyes)		245.00		284.00		264.0195		12.15557	
Outer inferior thickness		154 (308 eyes)		248.00		285.00		266.0455		11.37842	
Inner nasal thickness		154 (308 eyes)		290.00		333.00		306.7078		10.46059	
Inner temporal thickness		154 (308 eyes)		285.00		308.00		298.7597		5.64304	
Inner superior thickness		154 (308 eyes)		284.00		333.00		303.3442		12.14906	
Inner inferior thickness		154 (308 eyes)		278.00		320.00		296.9026		10.79323	
Central macular thickness		154 (308 eyes)		212.00		296.00		241.7532		17.30553	
Total volume		154 (308 eyes)		7.13		8.20		7.6056		0.33822	
Average RNFL		154 (308 eyes)		102.75		125.50		112.5601		5.19985	
Superior RNFL		154 (308 eyes)		101.00		133.00		122.2987		9.48968	
Inferior RNFL		154 (308 eyes)		125.00		153.00		138.5844		7.52226	
Nasal RNFL		154 (308 eyes)		90.00		137.00		116.3117		13.48614	
Temporal RNFL		154 (308 eyes)		58.00		98.00		73.0455		8.95036	

**Table 2:** **Table 2:** Difference of parameters according to gender

		*Gender*		*N*		*Mean*		*Std. deviation*		*Significance*	
Average macular thickness		Male		79 (158 eyes)		279.3082		7.33188		0.833	
		Female		75 (150 eyes)		279.5536		7.06081			
Outer nasal thickness		Male		79 (158 eyes)		283.3165		13.37386		0.697	
		Female		75 (150 eyes)		284.1333		12.55510			
Outer temporal thickness		Male		79 (158 eyes)		251.5316		5.35852		0.001	
		Female		75 (150 eyes)		255.7867		10.21564			
Outer superior thickness		Male		79 (158 eyes)		264.4684		12.71365		0.640	
		Female		75 (150 eyes)		263.5467		11.60533			
Outer inferior thickness		Male		79 (158 eyes)		267.0127		12.02934		0.280	
		Female		75 (150 eyes)		265.0267		10.63520			
Inner nasal thickness		Male		79 (158 eyes)		306.9114		10.18318		0.805	
		Female		75 (150 eyes)		306.4933		10.80962			
Inner temporal thickness		Male		79 (158 eyes)		298.3797		5.79191		0.393	
		Female		75 (150 eyes)		299.1600		5.49211			
Inner superior thickness		Male		79 (158 eyes)		303.7848		12.29621		0.646	
		Female		75 (150 eyes)		302.8800		12.05725			
Inner Inferior Thickness		Male		79 (158 eyes)		296.2911		10.69286		0.472	
		Female		75 (150 eyes)		297.5467		10.93259			
Central macular thickness		Male		79 (158 eyes)		242.0759		16.49302		0.813	
		Female		75 (150 eyes)		241.4133		18.22761			
Total volume		Male		79 (158 eyes)		7.6095		0.34302		0.884	
		Female		75 (150 eyes)		7.6015		0.33534			
Average RNFL		Male		79 (158 eyes)		112.2722		4.81138		0.482	
		Female		75 (150 eyes)		112.8633		5.59656			
Superior RNFL		Male		79 (158 eyes)		122.3544		9.75079		0.941	
		Female		75 (150 eyes)		122.2400		9.27193			
Inferior RNFL		Male		79 (158 eyes)		138.4177		7.46892		0.779	
		Female		75 (150 eyes)		138.7600		7.62436			
Nasal RNFL		Male		79 (158 eyes)		115.6329		14.21644		0.523	
		Female		75 (150 eyes)		117.0267		12.72789			
Temporal RNFL		Male		79 (158 eyes)		72.6835		7.74521		0.608	
		Female		75 (150 eyes)		73.4267		10.10509			

## DISCUSSION

Retinal thickness or macular thickness is important for diag nosis of early diabetic macular edema, cystoid macular edema, age-related macular degeneration and choosing appropriate management strategies in other cases of retinal diseases.^18^ Likewise, RNFL thickness assessment is important for detection of preperimetric glaucoma and damage to ganglion cell layer.^19-21^

**Table 3:** **Table 3:** Difference of parameters according to age

		*Age*		*N*		*Mean*		*Std. deviation*		*Significance*	
Average macular thickness		Less than 40 years		76 (152 eyes)		280.2600		7.18721		0.156	
		More than equal 40 years		78 (156 eyes)		278.6168		7.12273			
Outer nasal thickness		Less than 40 years		76 (152 eyes)		284.5921		12.69664		0.408	
		More than equal 40 years		78 (156 eyes)		282.8590		13.20933			
Outer temporal thickness		Less than 40 years		76 (152 eyes)		254.9211		9.03292		0.053	
		More than equal 40 years		78 (156 eyes)		252.3205		7.45461			
Outer superior thickness		Less than 40 years		76 (152 eyes)		264.7500		12.76153		0.463	
		More than equal 40 years		78 (156 eyes)		263.3077		11.57282			
Outer inferior thickness		Less than 40 years		76 (152 eyes)		266.3684		11.08253		0.729	
		More than equal 40 years		78 (156 eyes)		265.7308		11.72263			
Inner nasal thickness		Less than 40 years		76 (152 eyes)		308.0526		11.13301		0.116	
		More than equal 40 years		78 (156 eyes)		305.3974		9.65286			
Inner temporal thickness		Less than 40 years		76 (152 eyes)		298.9605		5.82510		0.664	
		More than equal 40 years		78 (156 eyes)		298.5641		5.49046			
Inner superior thickness		Less than 40 years		76 (152 eyes)		304.7632		12.61837		0.153	
		More than equal 40 years		78 (156 eyes)		301.9615		11.58755			
Inner inferior thickness		Less than 40 years		76 (152 eyes)		297.9474		11.41682		0.237	
		More than equal 40 years		78 (156 eyes)		295.8846		10.11875			
Central macular thickness		Less than 40 years		76 (152 eyes)		241.9868		16.05033		0.869	
		More than equal 40 years		78 (156 eyes)		241.5256		18.54844			
Total volume		Less than 40 years		76 (152 eyes)		7.6428		0.33884		0.179	
		More than equal 40 years		78 (156 eyes)		7.5694		0.33582			
Average RNFL		Less than 40 years		76 (152 eyes)		112.7599		5.23425		0.639	
		More than equal 40 years		78 (156 eyes)		112.3654		5.19251			
Superior RNFL		Less than 40 years		76 (152 eyes)		122.9079		9.38535		0.433	
		More than equal 40 years		78 (156 eyes)		121.7051		9.61345			
Inferior RNFL		Less than 40 years		76 (152 eyes)		138.6053		7.92478		0.973	
		More than equal 40 years		78 (156 eyes)		138.5641		7.15975			
Nasal RNFL		Less than 40 years		76 (152 eyes)		116.7632		13.23618		0.683	
		More than equal 40 years		78 (156 eyes)		115.8718		13.79663			
Temporal RNFL		Less than 40 years		76 (152 eyes)		72.7632		8.94408		0.701	
		More than equal 40 years		78 (156 eyes)		73.3205		9.00576			

Methods to assess macular thickness are slit-lamp biomicro scopy, fundus photography, fundus fuorescein angio-graphy and OCT. Among these, OCT alone provides quantitative assessment of macular thickness.^22-24^ OCT provi des for accurate assessment of details of retina and nerve fiber layer with high reproducibility and can be correlated well with clinical disease state.^25-29^ All the information thus collected needs to be analyzed and interpreted considering age, gender and racial differences.^9-11,13-17^

With various OCT machines available, we need to understand the normative data generated by both TD and SD-OCT machines before we can conclude about abnormalities and decide on management strategies. The color coding system of the analyzed report provides reasonable discrimi nation between normal and abnormal values.^12^ Our study done in central India provides for normative data of popu la tion visiting a tertiary care hospital and the data was collec ted using spectral domain OCT machine, which is a stan dard tool today.

In our study, the mean macular thickness (central 1 mm zone) with SD-OCT was 241.75 ± 17.3 microns. Compared with this, various studies done with time domain OCT repor ted macular thickness as 150 microns approximately.^2,30-33^

Massin P et al^34^ and Muscat S et al^28^ reported mean central macular thickness as 175 approximately, while Guedes V et al^33^ reported 210 microns as mean central macular thickness.

Ibrahim MA et al^35^ reported, the mean thickness was 188 mm (SD ± 20 mm) in normal eyes with TD-OCT and 266 mm (SD ± 21 mm) on SD-OCT. The mean thickness in the subfields N, S, T and I was: 266, 268, 255 and 267 mm, respectively, when measured by TD-OCT and 340, 340, 327 and 336 mm, respectively, when measured by SD-OCT. The difference in average thickness as measured by both OCT technologies was statistically significant in all subfields (p < 0.01). This difference in measurements could be attributed to the difference in measurement protocols used by various machines. Time domain OCT machines measure retinal thickness from IS/OS to ILM. The Topcon SD-OCT used in our study measures retinal thickness between the ILM and the posterior border of RPE. Factors other than segmentation algorithm (for example, density of sections, acquisition method, and acquisition speed) may contribute to differences in thickness measurements among devices.

Carineto P et al^36^ reported significant difference in macular thickness measured by SD-OCT (approximately 227 microns) *vs* TD-OCT (approximately 144 microns) in 40 healthy subjects. Grover S et al^37^ reported a difference of approximately 70 microns in the value of that mean central macular thickness between TD-OCT and SD-OCT. This increased measurement corresponds to the inclusion of the outer segment-RPE-Bruch's membrane complex by SD-OCT, which is relevant to studies using the newer SD-OCT for assessment of retinal thickness.

From above studies, it is evident that values of macular differ when measured using TD-OCT and SD-OCT. Thus, we conclude that while reviewing patients and retinal thickness, OCT machine, their protocols should be taken into account and values from different machines cannot be used for com parison or follow-up.

## RNFL

In our study, the mean macular volume was 7.6 ± 0.33 cu mm. On analysis of the RNFL thickness, we observed that the RN FL was thickest in the inferior quadrant (138.58) followed by superior (122.30) nasal (116.32) and temporal quadrant (73.04) (Table 1). The mean RNFL from ou r study was similar to the data available from other studies (Table 4).

Sony P et al^15^ in a cross-sectional study of 146 patients of OCT analysis on quadrant-wise analysis of the RNFL thickness, they observed that the RNFL was thickest in the inferior (132.34 ± 14.70 μ) and superior (131.09 ± 14.13 μ) quadrants. The thickness was lesser in nasal (85.93 ± 17.89 μ) and temporal (67.1 ± 12.77 μ) quadrants according to them, the difference between inferior and superior quadrants was not statistically significant suggesting that the ISNT rule does not apply to Indian eyes.

**Table 4:** **Table 4:** Comparative data of our study with RNFL values from other studies

		*Machine*		*N (no. of subjects)*		*Mean*		*Inferior*		*Superior*		*Nasal*		*Temporal*	
Current study		Topcon SD-OCT 3000		154		112.5 ± 5.1		138.5 ± 7.5		122.2 ± 9.4		116.3 ± 13.4		73.0 ± 8.9	
George Kampougeris et al^39^		SD-OCT + SLO (Optos, UK)		278		114.8 ± 13.3		134.5 ± 18.1		136.7 ± 18		107.2 ± 17.8		79.5 ± 15.3	
YM Tariq et al^40^		Cirrus SD-OCT		1521		99.4 ± 9.6		128.8 ± 17.1		124.7 ± 15.7		74.3 ± 12.8		69.9 ± 11.2	
Hirasawa et al^41^		Topcon SD-OCT		251		101.9 ± 8.4		125.5 ± 13.1		123.9 ± 13.6		79.6 ± 13.6		78.6 ± 13.3	
Bendschneider et al^42^		Spectralis SD		170		97.2 ± 9.7		123.7 ± 16.4		118.0 ± 14.5		76.4 ± 15		68.8 ± 11.1	
Huynh et al^43^		Stratus TD-OCT		2132		103.6 ± 10.6		128.3 ± 18.6		129.7 ± 17.5		82.0 ± 16.7		74.6 ± 12.8	

Kanamori et al^38^ in their study of 160 normal eyes showed slightly higher values than ours. They found that superior thickness (145.5 ± 19.6 μ*),* was maximum followed by inferior RNFL thickness (143.1 ± 19.5 μ), temporal (98.7 ± 20.8 μ*),* and last in nasal quadrant (92.6 ± 20.4 μ*).* Their observation also did not follow the previously described ISNT rule.

Ramakrishnan R et al^16^ in their study (Stratus OCT 3000; Carl Zeiss Ophthalmic Systems-Humphrey Division, Dublin, CA, USA) found that RNFL thickness for superior, inferior, nasal, and temporal quadrants were 138.2 ± 21.74 (95% CI: 134.3-142.1), 129.1 ± 25.67 (95% CI: 124.5-133.7), 85.71 ± 21 (95% CI: 81.9-89.5), and 66.38 ± 17.37 (95% CI: 63.3-69.5) μm, respectively. The mean RNFL thickness was highest in the superior quadrant followed by inferior, nasal, and temporal quadrants (ISNT rule not followed).

Table 4 gives summary of RNFL values and their compari son using various machines. It is clear from the data (Table 4) that RNFL obtained from various machines cannot be used interchangeably.

Seibold LK et al^44^ in their study of RNFL thickness from 40 normal subjects using 3 SD-OCT machines and one TD-OCT machine. The mean RNFL thickness was 106.6 ± 12.8 μm for Spectralis, 98.7 ± 10.9 μm for Cirrus, 112.8 ± 13.2 μm for RTVue and 110.1 ± 12.8 μm for Stratus. Despite high correlations, RNFL values are significantly different between instruments and should not be used interchangeably.

It is evident from review of literature that RNFL values obtained using TD- and SD-OCT show correlation but are different. They may not be comparable and should not be used for follow-up and comparison.^45-47^

Johnson DE et al^48^ studied RNFL thickness among 20 healthy volunteers using TD-OCT (Stratus) and SD-OCT (RTvue) and found that RNFL measurement with RTvue were thicker by approximately 20 microns as compared to values obtained with Stratus (TD-OCT), thus the technological difference does not allow direct comparison of data.

Lee ES et al^49^ in their study compared RNFL values of 108 open angle glaucoma patients and 46 controls using TD-OCT (Stratus) and SD-OCT (RTvue and Cirrus OCT). RNFL measurements were more with the RTvue, followed by the Stratus, and finally by the Cirrus OCT (p < 0.05). However, the tendency was reversed or no longer present in severe glaucomatous eyes and nasal quadrant maps. Thus, the study concluded that direct comparisons of RNFL thickness measurements among OCT instruments should not be done.

In our study, no significant variation was noted in mean central macular thickness and RNFL with age, gender and refractive error. Subjects with high refractive errors were excluded from the study as per protocol. Similar results were reported by Gobel et al^50^ and Sony P et al.^15^

The limitation of our study was relatively smaller sample size. Long-term studies with larger population base may be required to validate the results.

Thus, we highlight the fact that macular thickness values are different from TD-OCT and SD-OCT and are not comparable. However, RNFL values do not show such variation. To conclude our study gives data of macular thickness and RNFL in normal subjects using SD - OCT from central India which should form the basis for further studies.
